# Synchronous Display and Whiteboard-Like Freehand Writing App as Teaching Tool for Virtual Classroom amidst the Pandemic

**DOI:** 10.12688/f1000research.73531.2

**Published:** 2022-03-14

**Authors:** Daniel Lai, Lew Sook Ling, Ooi Shih Yin

**Affiliations:** 1 Faculty of Information Science and Technology, Multimedia University, Ayer Keroh, Melaka, 75450, Malaysia

**Keywords:** Mobile Interactive System, Virtual Classroom, TPACK, Educational Technology, COVID-19, Synchronous Display, Freehand Writing, Teaching Tool

## Abstract

**Purpose:** The research evaluates if teachers can achieve better teaching outcomes by using a proposed mobile interactive system (MIS) developed for this study as an additional approach to enhancing teachers’ proficiency in Technological Pedagogical and Content Knowledge (TPACK) in the virtual classroom.

**Background**: According to previous studies, teachers’ self-assessment on TPACK might be affected by their ego because they have autonomy over the students in the classroom. Some studies suggest that utilisation of an interactive whiteboard (IWB) promotes creativity in teaching and learning but that it is unsuitable for a virtual environment due to its large size and the high maintenance costs associated with owning one in a teacher’s residence. Besides, some studies also reveal that allowing the students to assess their teachers through TPACK  is able to reduce potential errors which might result from the TPACK self-assessment done by teachers.

**Methods:** Pre- and post- experiments were conducted with the developed MIS integrated into teaching process. Synchronous display (SD) and whiteboard-like freehand writing (WFW) were features of the MIS integrated into the experimental group. Questionnaires were distributed to the students, and a reflective measurement model was formed using SmartPLS and IBM SPSS Statistics.

**Findings**: Based on our findings, teachers’ Technological Content Knowledge had a significant positive effect on TPACK with the inclusion of MIS in online teaching. Predictive relevance was also evaluated through a Q ^2^ value to predict the endogenous construct of the constructed model. The Q ^2^ value was greater than zero, indicating that the model possesses a predictive relevance.

**Conclusion: **The integration of the developed MIS in the virtual classroom has a significant positive impact on the students’ academic performance relating to concept learning and knowledge acquisition of subject matter.

## Introduction

### Background

The outbreak of COVID-19 in 2020 caused a significant impact on the education sector. Pedagogy has changed drastically to cope with the pandemic and has many cases shifted from conventional in-classroom learning to online teaching. ^1^ The classical in-classroom teaching and learning is commonly practised to promote social interactions among teachers and students. ^2^ However, it is now unable to fit well into the current education environment due to the pandemic. ^3^ Because of this, the rapid evolution of technology will alter pedagogy where emerging technologies are accessible in education. ^4^

Additionally, teachers solely depend on the mouse and keyboard to conceptualise subject matter where conventional tools are prohibited in the virtual classroom. Hence, emerging technologies are excellent for content presentations in the virtual classroom. ^5^ From students’ perspective, attending lessons via synchronous learning video conferencing is preferred as it offers flexibility. Students are engaged in the virtual session as much as in the physical classroom. ^6^ However, the effectiveness of integrating technology in conducting online classes is questionable. In the Vasodavan et al. study (2020), the teachers are incompetent in incorporating technology effectively into their lessons and activity. ^7^ They do possess solid knowledge of the technology, but they can hardly fit this technology, especially into their teaching practices when it comes to integration. Hence, collaborative teaching is less effective when the teachers cannot identify the most appropriate technology to support their teaching practices.

Furthermore, technology integration in education never proposed an ultimate solution but offered an alternative. ^8^ Therefore, technology should not be the sole reason for being blamed as an instrumental issue. Besides, technology integration in the classroom leads to more issues or is somewhat problematic. Therefore, technology integration in education should emphasise alternativity instead of betterment in which the claim of “better technology” is very vague. The choice of technology should not consider only the latest trend but consider its functionality and suitability. There is no technology that can fit into all scenarios and criteria.

Moreover, the teachers are unprepared for the sudden transition to the online teaching approach. ^9^ Due to the lack of experience in online classes, they are not proficient in using unfamiliar technology to conduct classroom activities that they used to in the conventional class. For example, one of the mandatory technical skills required for the online class is video conferencing with the synchronous presentation in the virtual classroom. In addition, insufficient technical support from academic institutions leads to teachers’ incompetency in teaching with technology. ^10^ It somehow hinders the students’ understanding of the subject matters.

Additionally, the teachers with less experience and lower efficacy with information and communication technology (ICT) tend to undergo more negativity while teaching online classes. ^11^ Their willingness of adopting technology in their teaching was mainly due to their prior experience and perceived value towards the technology integration.

The adoption of the TPACK model aims to help teachers contemplate their knowledge domains (technological, pedagogical, and content knowledge) and the intersections of these to teach and engage students effectively. According to Hossain, Ying and Saha, TPACK positively influences higher education by making the lessons more interesting by observing learners’ needs. ^12^ From their studies, students have positive attitudes towards the TPACK model, in which their feedback can be addressed and used to construct lessons tailored to their needs. Moreover, teachers’ TPACK self-efficacy is enhanced in lesson planning with technology. ^13^

### Problem statement

Since the students are prohibited from entering their physical classes, teachers face challenges and difficulties when conducting online classes via the virtual classroom. ^14^ The practice of conducting classes through the virtual classroom sacrifices the benefits experienced in the conventional classroom environment. ^9^ It negatively affects the students’ knowledge acquisition of the subject matters from their teachers. Additionally, teachers are requested to prepare teaching materials and determine ways to present them in the virtual classroom. Although teachers nowadays are primarily aware of and understand the nature of technology and its functionality, they commonly face challenges in integrating the technology effectively into their pedagogy. ^7^ Thus, research questions are drawn as follows:

• Research Question 1: Does the integration of MIS into the virtual classroom enhance teachers’ self-efficacy from students’ perspectives in TPACK?
• Research Question 2: Does the integration of MIS into the virtual classroom enhance teachers’ efficacy in presenting and conceptualising teaching materials?

### Objectives

Since the introduction of the virtual classroom, it has become evident that it is essential to have a simple-to-use, tailored tool for assisting teachers in conducting online classes according to the current educational trend. Consequently, a mobile interactive system is proposed and developed to cope with the current trend. Furthermore, the introduction of the MIS offers teachers the ability to utilise the features of synchronous display (SD) and whiteboard-like freehand writing (WFW) features. Thus, the objectives are as follows:

1. Determine if the introduction of MIS features enhances teachers’ proficiency in TPACK from students’ perspectives.
2. Determine teachers’ efficacy in presenting and conceptualising teaching materials by constructing a model based on the integration of MIS with the adoption of the TPACK framework in the virtual classroom.

## Literature review

### Virtual classroom

In following the standard of procedures set by the government to reduce close contact, the transformation of the current education system is necessary. Thus, conducting classes through online platforms has become a new trend in education. ^15^ In the virtual classroom, classes are taught online synchronously via video conference. However, acquiring new skills and knowledge relating to communication, pedagogy, content, and structure is required due to distinctive differences in the teaching environment. ^16^ However, with the introductory of online courses, the curriculum planning gained more flexibility and openness. ^17^ Sometimes, students were assumed to adapt to technology instantaneously without any guidance and training because they were digital natives.

Nevertheless, that statement was debunked in Andrew’s study. He mentioned that the teachers and students were equally needed guidance on conducting effective communication and interaction in the online classes. From his findings, the teachers had a positive attitude regarding their interaction in the online class while the students had it otherwise. It was due to the different points of view of the teachers and students perceiving the interaction, social quality and teaching presence in the online class and blended learning. One of the teachers’ common external factors in curriculum design and planning was the lack of control. The syllabus of the subject matter could be pre-design according to particular learning outcomes. Hence, the teachers were not given complete authority in selecting more appropriate teaching approaches and content selections for teaching. The hybrid teaching approaches within one course could hinder the full potential of online learning as it sacrificed the flexibility in course design. The interaction between the teachers and students in the online class was a significant limitation due to the poorly designed online course.

### Interactive whiteboards

The combination of IWB with a virtual learning environment (VLE) exists with limited studies. According to Heemskerk, Kuipert, and Meijer’s study, implementing IWB with VLE can boost students’ motivation towards the subject matter. ^18^ However, the test given by teachers proves that it is ineffective to students’ learning outcomes even with the implementation of IWB and VLE. Furthermore, the IWB is an expensive and fragile device as it can be damaged easily, resulting in inconvenience when teaching with it, which does not justify long-term usage. ^19^ Besides, teachers still retain complete autonomy in the classroom, creating a teacher-centred environment while teaching with IWB ^20^

As a result, the students who learned in an IWB-based instruction classroom had more interest and motivation in learning. ^21^ Furthermore, integrating IWB in the classroom allowed the teachers to alter the teaching practices with more classroom activities that support their teaching academically. However, implementing interactive whiteboards in the classrooms did not improve students’ learning achievement. In short, IWB is not practical to be integrated into the virtual classroom because the IWB is difficult to maintain and not friendly for teachers who conduct classes at their residence during the pandemic.

### TPACK framework

As a teacher, one must be aware of and adapt to any changes, honing skills and knowledge to guide and expose students to state-of-art technologies in the virtual classroom. ^22^ However, insufficient teaching experience via virtual classrooms forces teachers to polish their skills and knowledge. Hence, they can cope with the current education phenomenon. As a result, the pedagogy and teaching materials presentation are elevated. ^23^ Therefore, the TPACK framework is adopted. It consists of seven elements, the three main elements being technological knowledge (TK), pedagogical knowledge (PK), content knowledge (CK), which are derived into four sub-elements pedagogical content knowledge (PCK), technological pedagogical knowledge (TPK), technological content knowledge (TCK), and TPACK. ^24^ Initially, the TPACK framework is teachers’ self-assessment about the thoroughness of their understanding of every element, including the teachers’ “know-how” in utilising the technology and integrating it into their pedagogy and teaching materials presentation. ^25^ However, assessing it from the teacher’s or student’s perspective brings different meanings. For example, the teachers’ perspective is viewed from the educator’s perspective, whereas the students’ perspective is viewed from the virtual classroom experience created by their teachers. ^26^ In short, the TPACK framework is vital because it helps assess the readiness of teachers’ knowledge and understanding in using technology integrated into their pedagogy and content presentation, both from the teachers’ and students’ perspectives.

## Methods

### Overview

**Figure 1.**
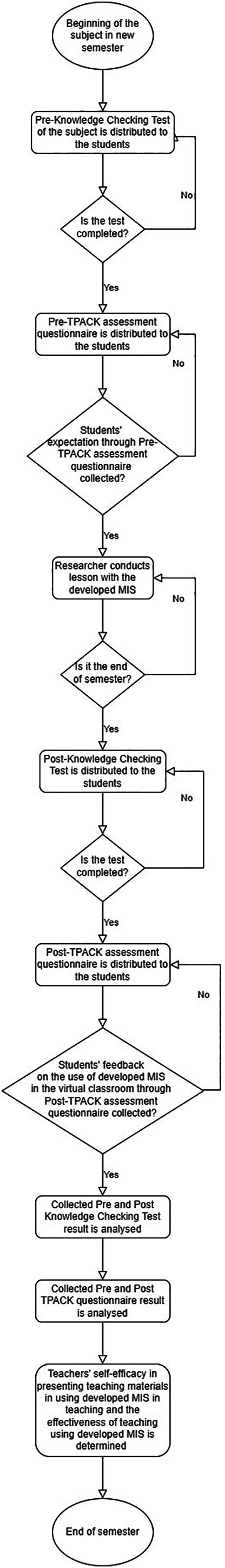
Flow chart of the integration of MIS and TPACK in the virtual classroom.

Rickles et al. (2017) mapped their research elements (Learning Environment Context and Learning Activity Context) with the adoption of the TPACK framework. ^27^ The Learning Activity influenced TK, PK, and CK, while the Learning Environment Context may affect all elements, including Learning Activity Context. Hence, the study proposes that the Learning Environment Context supports the relevancy of Learning Activity Context as complimentary learning. These two contexts are the keys of context-based learning (CBL), where the social aspect of learning and learning activity with solid context understanding in acquisition and processing knowledge are concerned. Moreover, CBL allows teachers to prepare early for teaching materials, making it easier to implement than problem-based learning, which is relatively time-consuming. Hence, the rapid change of teaching approach necessitated during the pandemic makes CBL more suitable for current phenomena in terms of content-oriented presentation. The overall flow in determining teachers’ self-efficacy in presenting and conceptualising teaching materials using MIS is illustrated in Figure 1. The experiment is designed based on a quasi-experiment. It consists of pre-test and post-test of an experimental group. The pre-test and post-test of the knowledge checking test share similar intention of the test conducted by Mustafa and Meric on learning achievement. ^28^ As for the pre-test and post-test of TPACK, assessments are conducted to observe whether the difference is significant regarding the teachers’ improvement in TPACK knowledge domains’ understanding. ^29^

Besides, a pilot study was conducted to gather feedback and insights from the students and experts before conducting the actual survey. The findings of the pilot study survey indicated that the users were showing a positive attitude towards the integration of the proposed MIS as learning media.

### Participants

Cluster sampling was implemented. Students who experienced online learning with the integration of the MIS features were chosen as the cluster because of its capability to reduce sample bias. This data collection method provides a better representation of populations. ^30^ Online questionnaires were distributed to 45 Malaysian private university students enrolling in “Knowledge Management”. The chosen group was taught with MIS integrated into their online classes.

### Procedure

The experiment was conducted from the start till the end of the semester (3 months). This study is a quasi-experimental one-group with the pre-and-post-tests. The MIS is tailored for online classes with two major features: SD and WFW. The questionnaires were created to adopt the TPACK framework and prepared in two sets specifically for pre-test and post-test through Google Form. Besides, conducting TPACK self-assessment from teachers’ perspectives may lead to biases. The assessment can be inaccurate as assessing themselves is not convincing enough to reflect if their understanding is up to par. ^31^ Thus, to reduce the bias from the teacher’s self-assessment, the student’s perspective on TPACK should be considered instead. ^32^ The collected data is trimmed and analysed using SmartPLS software v3.3.2.R is an open-source alternative ( R Project for Statistical Computing, RRID:SCR_001905).

## Results

**Figure 2.**
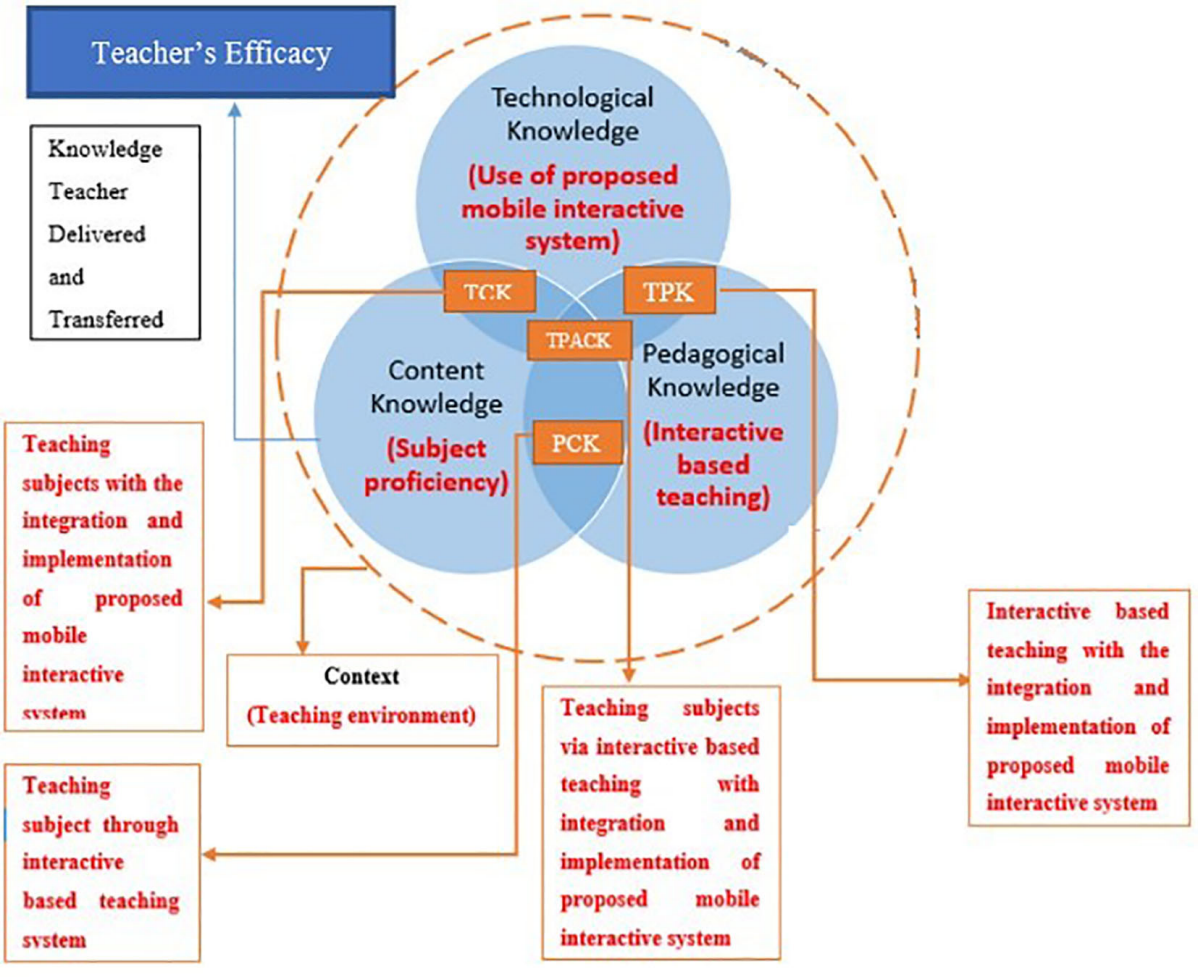
Adoption of TPACK framework with MIS teacher’s efficacy mapped.

In this study, the adoption of the TPACK framework is proposed. The teacher’s efficacy mapped in conjunction with the integration of MIS in the virtual classroom is illustrated in Figure 2.

**Figure 3.**
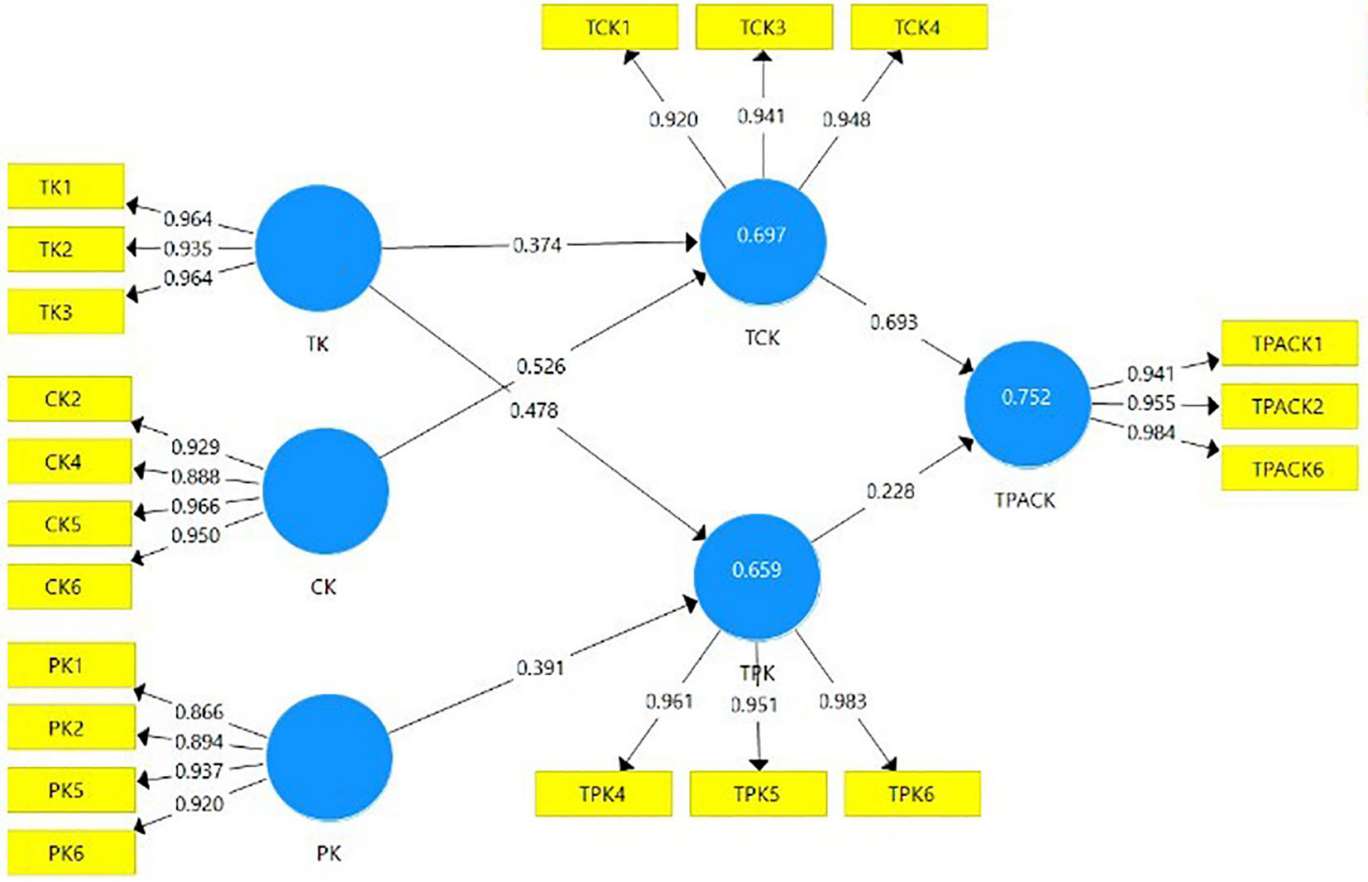
Proposed model with the adoption of TPACK framework.

Through PLS-SEM, the proposed model is constructed in Figure 3 based on adopting the TPACK framework. Moreover, the proposed model is constructed as a reflective Measurement Model.

Firstly, after running Partial Least Squared Algorithm, the constructs’ outer loadings indicators are observed in Table 1. The loadings for the construct indicators are > 0.7.

**Table 1.** Indicators of the constructs’ outer loadings.

	CK	PK	TCK	TK	TPACK	TPK
CK2	0.929					
CK4	0.888					
CK5	0.966					
CK6	0.950					
PK1		0.866				
PK2		0.894				
PK5		0.837				
PK6		0.820				
TCK1			0.920			
TCK3			0.941			
TCK4			0.948			
TK1				0.964		
TK2				0.935		
TK3				0.964		
TPACK1					0.941	
TPACK2					0.955	
TPACK6					0.984	
TPK4						0.961
TPK5						0.951
TPK6						0.983

Secondly, the construct reliability and validity are examined, as shown in Table 2. The threshold value for the composite reliability (CR) is > 0.70, while the threshold value for the average variance extracted (AVE) is > 0.5. ^34^^,^^35^ In this study, the CR and AVE are fulfilling the threshold requirements as the values of CR are > 0.9, and the values of AVE are > 0.75. The CR values indicate good reliability, and AVE values indicate good validity.

**Table 2.** Construct reliability and validity.

	Cronbach’s alpha	rho A	Composite reliability	Average variance extracted (AVE)
CK	0.950	0.952	0.964	0.871
PK	0.927	0.947	0.947	0.819
TCK	0.930	0.933	0.955	0.877
TK	0.951	0.957	0.968	0.911
TPACK	0.957	0.962	0.972	0.922
TPK	0.963	0.965	0.976	0.932

Thirdly, the discriminant validity of the model is inspected. The Heterotrait-Monotrait Ratio (HTMT) is preferred because of its stringent measures with sensitivity rates of 97%-99%. ^35^ On the other hand, the Fornell-Larcker Criterion is insensitive with a rate of 20.82%. ^35^ Furthermore, the HTMT values of this study are above 0.85 and less than 0.90 ( Table 3). Therefore, it satisfied the HTMT threshold. Hence, the discriminant validity between relative constructs is established. Next, the significance and relevance of the structural model in this study are assessed, as shown in Table 4. First, a One-Tailed test Bias-corrected and accelerated bootstrap (BCA) with bias and skewness adjusted are conducted. As research hypotheses are directional, the bootstrapping is with 5000 subsamples. Then, the path coefficients are obtained and further examined. The path coefficients values are closer to +1, representing strong positive relationships between latent constructs. Additionally, the path coefficient values should be at the least 0.05 significant level.

**Table 3.** Heterotrait-Monotrait Ratio (HTMT) for discriminant validity.

	CK	PK	TCK	TK	TPACK	TPK
CK						
PK	0.834					
TCK	0.861	0.781				
TK	0.790	0.850	0.795			
TPACK	0.846	0.813	0.900	0.756		
TPK	0.894	0.879	0.730	0.798	0.735	

**Table 4.** Path coefficients.

	Original sample mean	Standard deviation (STDEV)	P-values
CK -> TCK	0.526	0.206	0.005 ***
PK -> TPK	0.391	0.229	0.044 ***
TCK -> TPACK	0.693	0.143	0.000 ***
TK -> TCK	0.374	0.182	0.020 ***
TK -> TPK	0.478	0.224	0.017 ***
TPK -> TPACK	0.228	0.181	0.104

*** Significant at 0.05.

Next, the R ^2^ and R ^2^ adjusted values are shown in Table 5 are considered substantial. ^33^

**Table 5.** R ^2^ and R ^2^ adjusted for calculating effect size.

	R square	R square adjusted
TCK	0.697	0.683
TPACK	0.752	0.741
TPK	0.659	0.643

The effect size is calculated using the formula below:

$$f^{2}=\;\frac{R^{2}\;\text{included}-R^{2}\;\text{excluded}}{1-R^{2}\;\text{included}}$$

The results in Table 6 indicate that the TPK (0.109) has a negligible effect in producing the R ^2^ for TPACK. However, the TCK (1.008) illustrates that it has a significant impact on producing the R ^2^ for TPACK.

**Table 6.** Effect size (f ^2^).

	**TPACK**
TCK	1.008
	**TPACK**
TPK	0.109

The results for the blindfolding are in the rightmost column, which is shown in Table 7. The predictive relevance Q ^2^ of TCK has a value of 0.598, TPK has a value of 0.579, and TPACK has a value of 0.675. These values indicate that the model has predictive relevance based on the three endogenous constructs in which the values of Q ^2^ are considerably above zero.

**Table 7.** Construct cross-validated redundancy (CVR) Using blindfolding procedure.

	SSO	SSE	Q ^2^ (= 1 − SSE/SSO)
CK	188.000	188.000	
PK	188.000	188.000	
TCK	141.000	56.685	0.598
TK	141.000	141.000	
TPACK	141.000	47.767	0.675
TPK	141.000	59.319	0.579

As shown in Table 8, a paired-samples T-test was performed to identify the students’ improvement on their understanding of the subject matter with the integration of developed MIS. The mean score difference of −3.289 shows that students’ understanding of the subject matter is improved. The p-value of 0.00000003178 denotes that integrating the developed MIS in the virtual classroom positively affects the students’ academic performance. The improvement over the pre- and post-knowledge checking tests are substantially significant.

**Table 8.** Paired-samples T test.

Paired difference								
	Mean	Std. deviation	Std. error mean	95% confidence interval of the difference		t	df	Sig (2-tailed)
				Lower	Upper			
Pre Score – Post Score	−3.289	2.912	.472	−4.247	−2.322	−6.963	37	.00000003178

## Discussion

The involvement of technology in education acts as a catalyst in transforming the conventional classroom into the virtual classroom. The extensive involvement of technology in the current education trend prompted the adoption of the TPACK framework in this study. However, educational tools such as interactive whiteboards are not practical in the virtual classroom, although they offer teachers various useful features. ^36^ Therefore, the developed MIS is integrated into online teaching as a complementary pedagogy that assists the teachers in presenting teaching materials. Furthermore, the proposed model is constructed based on adopted TPACK framework elements. The consideration of students’ perspective TPACK is mainly a result of the potential bias and misconceptions raised by the teachers’ self-assessment on TPACK proficiency in the previous study. ^29^ Instead, this study addressed and mitigated those issues by gaining feedback and insights from the students’ perspectives. The students’ feedback gives a more in-depth insight allowing teachers to adjust their teaching practices and content presentation of subject matter. From the result of the path coefficient, we can see that a teacher with great understanding in transforming the content delivering it to the students using developed MIS possess the ability to enhance classroom activities which reflects their proficiency in TPACK. This phenomenon explains the importance of technology selection in which the technology can be utilised. With the proper tool, the teachers can enhance the teaching and learning experience and overcome the technology integration issues faced in the past study. ^7^ A well-fitted technology for teaching practices improves students’ performance academically. From the result of the paired-samples T-test, the mean difference for the pre-score and post-score indicates that the students are improved academically from concept and knowledge acquisitions. The outcome also illustrates that the teachers’ proficiency in TPACK is closely related to the effectiveness of the integration of developed MIS in the virtual classroom.

Additionally, the developed MIS with SD and WFW is tailored to assist teachers in conducting online classes. Familiarised teaching experience is replicated especially teaching materials presentation. Students’ feedback plays a vital role in determining the proficiency and self-efficacy of the teachers. Therefore, it is essential to address more insights into integrating technology into the virtual classroom than teachers’ self-assessment. In this study, teachers’ integration of MIS into the virtual classroom affects the TCK and TPACK. It indicates that the thoroughness of teachers’ understanding of the technology in presenting their teaching materials determines their students’ learning experience in the virtual classroom.

In short, the technology integration in this study had proven that the proficiency of a teacher’s knowledge about TPACK domains and sub-domains is essential. It affects the student’s learning outcomes and academic achievements. The proposed MIS in the online class positively impacts the teaching and learning experience. In the virtual classroom, the teachers have limited choices of tools to present their content of the subject matter. On top of that, their efficacy in using technology is also varied. Hence, the proposed MIS gives the teachers an alternative way of presenting the content of the subject matter in the virtual classroom. In the meantime, their teaching techniques and practices were restructured and improved with the integration of the proposed MIS.

## Conclusions

This study demonstrates the proposed structural model with the adoption of the TPACK framework and the integration of the proposed MIS with SD and WFW, assisting teachers in improving the online class experience with alternative teaching materials. Furthermore, the proposed structural model as a reflective measurement is constructed by adopting the TPACK framework as the fundamental. Looking at the relative importance of the exogenous constructs in predicting the dependent construct (TCK), it is evident that CK is the most crucial predictor followed by TK. The factor of TCK has a strong effect on TPACK. As for R ^2^, the value for TCK, TPK, and TPACK is considered substantial. The effect size for the TCK is considerably large. The student’s understanding of the subject matter is improved significantly with the integration of developed MIS by their teacher in the virtual classroom. In contrast with previous studies, the integration of developed MIS is more in accord with the virtual classroom, significantly enhancing teaching materials. The integration of the developed MIS in the virtual classroom has a significant positive impact on the students’ academic performance relating to concept and knowledge acquisition of subject matter.

### Limitation

There were several limitations faced in this study. One of the most significant limitations is the number of respondents for the survey distributed. In addition, the students who participated in the experiment were pre-assigned and divided into smaller tutorial groups. Hence, the sample size is considerably small. Consequently, it might affect the accuracy and reliability of the findings.

The following limitation of this study is the teachers’ choice of teaching approach and curriculum design. In addition, the teaching materials of the subject matter used in this study were standardised, where the content of the subject matter is prohibited from major editing. Therefore, the only factor that the teachers controlled was their pedagogy applied.

### Future enhancement

This study can be further improved with a larger sample size with greater accuracy and reliability. It also can mitigate the bias led by smaller sample size. Besides, allowing the teachers to prepare the teaching materials and the content of the subject matter on their own gives them more flexibility. The ability to design their curriculum also allows them to keep their teaching materials relevant to the latest trend and, most importantly, tailored according to their students’ needs and feedback. It also reflects a teacher’s efficacy in integrating technological tools into their teaching while their proficiency in TPACK elements is also addressed in a clearer picture.

### Ethics and consent

All the procedures performed in this study involving human participants adhered to the ethical policies of the Multimedia University as approved by the Technology Transfer Office of Multimedia University under ethical approval number: EA0732021.

Written consent was also obtained from all individual participants involved in the study.

## Data availability

Zenodo: TPACK MIS Dataset. https://doi.org/10.5281/zenodo.5744892. ^37^

This project contains the following underlying data:

• Dataset TPACK MIS 01122021.xlsx (The file contains two sheets. The first one contains the indicators of seven variables; TK, PK, CK, PCK, TPK, TCK, and TPACK used for framework analysis. The second sheet includes the results of students’ performance before and after using the MIS as pre-test and post-test).

Data are available under the terms of the Creative Commons Attribution 4.0 International license (CC-BY 4.0).
