# Supplementary material for: Synchronous Display and Whiteboard-Like Freehand Writing App as Teaching Tool for Virtual Classroom amidst the Pandemic
Source: F1000Res. 2022 Mar 14;10:1308. Originally published 2021 Dec 22. [Version 2] doi: 10.12688/f1000research.73531.2 (PMC8948440; doi:10.12688/f1000research.73531.2)
Supplement: Peer review history

# Peer review: Reviewer response for version 2

**DOI:** 10.5256/f1000research.122054.r127435

**Published:** 2022-03-29

**Authors:** Siti Hadiati Nugraini

**Affiliations:** 1 IT Master of Study Program, Faculty of Computer Science, Dian Nuswantoro University, Semarang, Indonesia

This article was approved because revisions to methods have been made on participants and the flow chart symbols of the integration of MIS and TPACK in the virtual classroom.

Is the work clearly and accurately presented and does it cite the current literature?

Yes

If applicable, is the statistical analysis and its interpretation appropriate?

Yes

Are all the source data underlying the results available to ensure full reproducibility?

Yes

Is the study design appropriate and is the work technically sound?

Partly

Are the conclusions drawn adequately supported by the results?

Yes

Are sufficient details of methods and analysis provided to allow replication by others?

Yes

Reviewer Expertise:

Multimedia Innovative of Learning

I confirm that I have read this submission and believe that I have an appropriate level of expertise to confirm that it is of an acceptable scientific standard.

# Peer review: Reviewer response for version 2

**DOI:** 10.5256/f1000research.122054.r127436

**Published:** 2022-03-24

**Authors:** Uchenna Cyril Eze

**Affiliations:** 1 Division of Business and Management, Beijing Normal University & Hong Kong Baptist University United International College (BNU-HKBU United International College), Zhuhai, China

Revised version is fine. No further comments. My concerns were addressed.

Is the work clearly and accurately presented and does it cite the current literature?

Yes

If applicable, is the statistical analysis and its interpretation appropriate?

Yes

Are all the source data underlying the results available to ensure full reproducibility?

Yes

Is the study design appropriate and is the work technically sound?

Yes

Are the conclusions drawn adequately supported by the results?

Partly

Are sufficient details of methods and analysis provided to allow replication by others?

Partly

Reviewer Expertise:

Information systems, MIS, e-business, e-learning, teaching and learning pedagogy, KM, marketing, business ethics

I confirm that I have read this submission and believe that I have an appropriate level of expertise to confirm that it is of an acceptable scientific standard.

# Peer review: Reviewer response for version 1

**DOI:** 10.5256/f1000research.77186.r118454

**Published:** 2022-02-07

**Authors:** Uchenna Cyril Eze

**Affiliations:** 1 Division of Business and Management, Beijing Normal University & Hong Kong Baptist University United International College (BNU-HKBU United International College), Zhuhai, China

I read the manuscript titled “Synchronous Display and Whiteboard-Like Freehand Writing App as Teaching Tool for Virtual Classroom amidst the Pandemic” with great interest.

 It explored the integration of technology pedagogy and knowledge content in a virtual learning environment. Specifically, it evaluates the effectiveness of teaching and learning that combines educational technology and knowledge content during the covid-19 pandemic period. The content in the manuscript was presented well, concise, highlighting relevant literature, with appropriate research design, analysis, and well-organized.

 However, the manuscript lacks detail. I am not sure if this was because of the journal’s requirements or the authors’ choice. My view is that this manuscript will benefit from additional relevant literature to provide more detailed perspective of prior research in the area. More synthesis of prior findings and how those findings fit into the findings in this manuscript. The authors could provide additional links between the discussions/findings in this manuscript to teaching and learning frameworks. This manuscript will also benefit from a detailed discussion of the outcome of the analysis, including clear contributions to teaching and learning, and to theory/literature (if any). There should be sections of the discussion devoted to the limitations of the study and potential agenda for future research. These will help to strengthen the quality, relevance, and currency of this manuscript.

 The manuscript, overall, appears well-written, however, there is the need for the authors to conduct a check on the writing quality and style to improve the overall readability and presentation in the manuscript.

Is the work clearly and accurately presented and does it cite the current literature?

Yes

If applicable, is the statistical analysis and its interpretation appropriate?

Yes

Are all the source data underlying the results available to ensure full reproducibility?

Yes

Is the study design appropriate and is the work technically sound?

Yes

Are the conclusions drawn adequately supported by the results?

Partly

Are sufficient details of methods and analysis provided to allow replication by others?

Partly

Reviewer Expertise:

Information systems, MIS, e-business, e-learning, teaching and learning pedagogy, KM, marketing, business ethics

I confirm that I have read this submission and believe that I have an appropriate level of expertise to confirm that it is of an acceptable scientific standard, however I have significant reservations, as outlined above.

# Response

**Published:** 2022-03-02

**Authors:** Sook Ling Lew

**Affiliations:** Multimedia University, Malaysia

**Literature Review**

 Relevant literature was provided in the Literature Review section as follows:

 Virtual Classroom

1. However, with the introductory of online courses, the curriculum planning gained more flexibility and openness (Blain 2019). Sometimes, students were assumed to adapt to technology instantaneously without any guidance and training because they were digital natives. However,  that statement was debunked in Andrew’s study. He mentioned that the teachers and students were equally needed guidance on conducting effective communication and interaction in the online classes. From his findings, the teachers had a positive attitude regarding their interaction in the online class while the students had it otherwise. It was due to the different points of view of the teachers and students perceiving the interaction, social quality and teaching presence in the online class and blended learning. One of the teachers’ common external factors in curriculum design and planning was the lack of control. The syllabus of the subject matter could be pre-design according to particular learning outcomes. Hence, the teachers were not given complete authority in selecting more appropriate teaching approaches and content selections for teaching. The hybrid teaching approaches within one course could hinder the full potential of online learning as it sacrificed the flexibility in course design. The interaction between the teachers and students in the online class was a significant limitation due to the poorly designed online course.

Interactive Whiteboard

1. As a result, the students who learned in an IWB-based instruction classroom had more interest and motivation in learning (Shi et al., 2018). Integrating IWB in the classroom allowed the teachers to alter the teaching practices with more classroom activities that support their teaching academically. However, the implementation of interactive whiteboards in the classrooms did not improve the student’s learning achievement.

**Discussion**

 The proposed research contribution was highlighted with references in the Discussion section as follows

1. However, educational tools such as interactive whiteboards are not practical in the virtual classroom, although they offer teachers various useful features (E.Science, 2020).
2. Instead, this study addressed and mitigated those issues by gaining feedback and insights from the students’ perspectives. The students’ feedback gives a more in-depth insight allowing teachers to adjust their teaching practices and content presentation of subject matter.
3. This phenomenon explains the importance of technology selection in which the technology can be utilised (Vasodavan et al., 2019). With the proper tool, the teachers can enhance the teaching and learning experience and overcome the technology integration issues faced in the past study. A well-fitted technology for teaching practices improves students’ performance academically.
4. In short, the technology integration in this study had proven that the proficiency of a teacher’s knowledge about TPACK domains and sub-domains is essential. It affects the student’s learning outcomes and academic achievements. The use of the proposed MIS in the online class positively impacts the teaching and learning experience. In the virtual classroom, the teachers have limited choices of tools to present their content of the subject matter. On top of that, their efficacy in using technology is also varied. Hence, the proposed MIS gives the teachers an alternative way of presenting the content of the subject matter in the virtual classroom. In the meantime, their teaching techniques and practices were restructured and improved with the integration of the proposed MIS.

**Limitations**

 Limitations section was added with the content as follows:

- There were several limitations faced in this study. One of the most significant limitations is the number of respondents for the survey distributed. In addition, the students who participated in the experiment were pre-assigned and divided into smaller tutorial groups. Hence, the sample size is considerably small. Consequently, it might affect the accuracy and reliability of the findings.
- The following limitation of this study is the teachers’ choice of teaching approach and curriculum design. In addition, the teaching materials of the subject matter used in this study were standardised, where the content of the subject matter is prohibited from major editing. Therefore, the only factor that the teachers controlled was their pedagogy applied.

**Future Enhancement**

 Future Enhancement section was added with the content as follows:

- This study can be further improved with a larger sample size with greater accuracy and reliability. It also can mitigate the bias led by a smaller sample size. Besides, allowing the teachers to prepare the teaching materials and the content of the subject matter on their own gives them more flexibility. The ability to design their curriculum also allows them to keep their teaching materials and contents of the subject matter relevant to the latest trend and, most importantly, tailored according to their students’ needs and feedback. It also reflects a teacher’s efficacy in integrating technological tools into their teaching while their proficiency in TPACK elements is also addressed in a clearer picture.

# Peer review: Reviewer response for version 1

**DOI:** 10.5256/f1000research.77186.r118453

**Published:** 2022-02-02

**Authors:** Siti Hadiati Nugraini

**Affiliations:** 1 IT Master of Study Program, Faculty of Computer Science, Dian Nuswantoro University, Semarang, Indonesia

APPROVED WITH MINOR RESERVATIONS

**Introduction - Background Section:** 

 The study is relevant and acceptable. However, the study should examine other related research works.

**Method Section – Overview Section:**

**Needs Analysis.**

 The first step taken by researchers, before designing learning media that will be used by users, is usually to carry out the stages of analyzing user needs. This is done by making a needs analysis questionnaire which is given to users of learning media that will be used in research. So, it is hoped that the learning media used in this study are really needed by users.

**Overview, Figure 1 (Page 4)**

 "Beginning of the subject in new semester" on Figure 1, usually use “Ellipse Symbol”.

**Note:**

 The elliptical shape symbol on the flowchart can be used to “START” and “END” activities.

**Methods - Participants: (Page 4)**

 It is possible to include the number of populations involved in this study. The article only mentions the sample, the population size has not been explained.

 I confirm that I have read this submission and believe that I have an appropriate level of expertise to confirm that it is of an acceptable scientific standard, with reservations, as outlined above.

Please see here for a further diagram to help clarify the reviewer's suggestion about Figure 1.

Is the work clearly and accurately presented and does it cite the current literature?

Yes

If applicable, is the statistical analysis and its interpretation appropriate?

Yes

Are all the source data underlying the results available to ensure full reproducibility?

Yes

Is the study design appropriate and is the work technically sound?

Partly

Are the conclusions drawn adequately supported by the results?

Yes

Are sufficient details of methods and analysis provided to allow replication by others?

Yes

Reviewer Expertise:

Multimedia Innovative of Learning

I confirm that I have read this submission and believe that I have an appropriate level of expertise to confirm that it is of an acceptable scientific standard, however I have significant reservations, as outlined above.

# Response

**Published:** 2022-03-02

**Authors:** Sook Ling Lew

**Affiliations:** Multimedia University, Malaysia

**Introduction - Background Section:**

 Related research works were examined and updated in the Introduction – Background section as follows:

1. In the Vasodavan et al. study (2020), the teachers are incompetent in incorporating technology effectively into their lessons and activity. They do possess solid knowledge of the technology, but they can hardly fit this technology, especially into their teaching practices when it comes to integration. Hence, collaborative teaching is less effective when the teachers cannot identify the most appropriate technology to support their teaching practices. Furthermore, technology integration in education never proposed an ultimate solution but offered an alternative (Castañeda and Selwyn, 2018. Therefore, technology should not be the sole reason for being blamed as an instrumental issue. Besides, technology integration in the classroom leads to more issues or is somewhat problematic. Therefore, technology integration in education should emphasise alternativity instead of betterment in which the claim of “better technology” is very vague. The choice of technology should not consider only the latest trend but consider its functionality and suitability. There is no technology that can fit into all scenarios and criteria.
2. Moreover, the teachers are unprepared for the sudden transition to the online teaching approach. Due to the lack of experience in online classes, they are not proficient in using unfamiliar technology to conduct classroom activities that they used to in the conventional class. For example, one of the mandatory technical skills required for the online class is video conferencing with the synchronous presentation in the virtual classroom. It somehow hinders the students’ understanding of the subject matters. Furthermore, the teachers wilh less experience and lower efficacy with information and communication technology (ICT) tend to undergo more negativity while teaching online classes (van der Spoel et al., 2020). Their willingness of adopting technology in their teaching was mainly due to their prior experience and perceived value towards the technology integration.

**Method Section – Overview Section:**

 The proposed mobile interactive system integrated into the study had great similarity with the conventional whiteboard and the virtual whiteboard provided by some online social platforms. In addition, the teachers used the on-screen writing feature of the proposed system as an alternative for the conventional whiteboard. Hence, the teachers could replicate the conventional teaching and learning experience into the virtual classroom.

 Hence, additional information was added in the study at Method – Overview as follows:

- Besides, a pilot study was conducted to gather feedback and insights from the students and experts before conducting the actual survey. The findings of the pilot study survey indicated that the users were showing a positive attitude towards the integration of the proposed MIS as learning media. 

**Overview, Figure 1 (Page 4)**

 Figure 1 was updated in the Overview section with Ellipse Shape Symbol for ‘Beginning of the subject in new semester’ instead of rounded-edge rectangle shape.

**Methods - Participants: (Page 4)**

 The population info regarding the participants of the study was updated in the Method – Participants section as follows:

- Online questionnaires are distributed to 45 Malaysian private university students enrolling in “Knowledge Management”.

# Peer review: Reviewer response for version 1

**DOI:** 10.5256/f1000research.77186.r118452

**Published:** 2022-01-05

**Authors:** Dalbir Singh

**Affiliations:** 1 Universiti Kebangsaan Malaysia, Bangi, Malaysia

**Introduction - Background Section:** 

 The study is relevant and acceptable. However, the need for such a study should be precisely justified. The study should examine other related research works. A critical review of approaches exploited in other studies should be performed to enhance the overall contribution further.  

**Introduction - Problem Statement Section:**

 Add more references that support the main issue in the study. Justify the selection of the main issue compared to other issues pertaining to teaching tools for a virtual classroom.

**Method Section – Overview Section:**

 Justify the selection and formulation of the approach as illustrated in Figure 1. Review relevant studies that use a similar approach.

**Discussion Section:**

 Review the research contribution against other research works. How different is the proposed research contribution? Add references.

Is the work clearly and accurately presented and does it cite the current literature?

Partly

If applicable, is the statistical analysis and its interpretation appropriate?

Yes

Are all the source data underlying the results available to ensure full reproducibility?

Yes

Is the study design appropriate and is the work technically sound?

Yes

Are the conclusions drawn adequately supported by the results?

Partly

Are sufficient details of methods and analysis provided to allow replication by others?

Yes

Reviewer Expertise:

Human-Computer Interaction, Impact Study & Strategic Planning, E-Learning Technology, Quality Assurance

I confirm that I have read this submission and believe that I have an appropriate level of expertise to confirm that it is of an acceptable scientific standard, however I have significant reservations, as outlined above.

# Response

**Published:** 2022-03-02

**Authors:** Sook Ling Lew

**Affiliations:** Multimedia University, Malaysia

**Introduction - Background Section:** 

 Related research works were examined as follows:

1. In the Vasodavan et al. study (2020), the teachers are incompetent in incorporating technology effectively into their lessons and activity. They do possess solid knowledge of the technology, but they can hardly fit this technology, especially into their teaching practices when it comes to integration. Hence, collaborative teaching is less effective when the teachers cannot identify the most appropriate technology to support their teaching practices. Furthermore, technology integration in education never proposed an ultimate solution but offered an alternative (Castañeda and Selwyn, 2018. Therefore, technology should not be the sole reason for being blamed as an instrumental issue. Besides, technology integration in the classroom leads to more issues or is somewhat problematic. Therefore, technology integration in education should emphasise alternativity instead of betterment in which the claim of “better technology” is very vague. The choice of technology should not consider only the latest trend but consider its functionality and suitability. There is no technology that can fit into all scenarios and criteria.
2. Moreover, the teachers are unprepared for the sudden transition to the online teaching approach. Due to the lack of experience in online classes, they are not proficient in using unfamiliar technology to conduct classroom activities that they used to in the conventional class. For example, one of the mandatory technical skills required for the online class is video conferencing with the synchronous presentation in the virtual classroom. It somehow hinders the students’ understanding of the subject matters. Additionally, the teachers wilh less experience and lower efficacy with information and communication technology (ICT) tend to undergo more negativity while teaching online classes (van der Spoel et al., 2020). Their willingness of adopting technology in their teaching was mainly due to their prior experience and perceived value towards the technology integration.

**Introduction - Problem Statement Section:**

 Supportive references were added in the Introduction – Problem Statement section as follows:

1. The practice of conducting classes through the virtual classroom sacrifices the benefits experienced in the conventional classroom environment. It negatively affects the students’ knowledge acquisition of the subject matters from their teachers (Coman et al., 2020).
2. Although teachers nowadays are primarily aware of and understand the nature of technology and its functionality, they commonly face challenges in integrating the technology effectively into their pedagogy (Vasodavan et al., 2019).

**Method Section – Overview Section:**

 The selection and formulation of the approach illustrated in Figure 1 were justified  in the Methods – Overview section as follows:

- The experiment is designed based on a quasi-experiment. It consists of pre-test and post-test of an experimental group. The pre-test and post-test of the knowledge checking test share similar intentions of the test conducted by Mustafa and Meric (2019) on learning achievement. As for the pre-test and post-test of TPACK, assessments are conducted to observe whether the difference is significant regarding the teachers’ improvement in TPACK knowledge domains’ understanding (Kartal and Dilek, 2021).

**Discussion Section:**

 The proposed research contribution was highlighted with references in the Discussion section as follows:

1. However, educational tools such as interactive whiteboards are not practical in the virtual classroom, although they offer teachers various useful features (E.Science, 2020).
2. Instead, this study addressed and mitigated those issues by gaining feedback and insights from the students’ perspectives. The students’ feedback gives a more in-depth insight allowing teachers to adjust their teaching practices and content presentation of subject matter.
3. This phenomenon explains the importance of technology selection in which the technology can be utilised. With the proper tool, the teachers can enhance the teaching and learning experience and overcome the technology integration issues faced in the past study (Vasodavan et al., 2019). A well-fitted technology for teaching practices improves students’ performance academically.
4. In short, the technology integration in this study had proven that the proficiency of a teacher’s knowledge about TPACK domains and sub-domains is essential. It affects the student’s learning outcomes and academic achievements. The use of the proposed MIS in the online class positively impacts the teaching and learning experience. In the virtual classroom, the teachers have limited choices of tools to present their content of the subject matter. On top of that, their efficacy in using technology is also varied. Hence, the proposed MIS gives the teachers an alternative way of presenting the content of the subject matter in the virtual classroom. In the meantime, their teaching techniques and practices were restructured and improved with the integration of the proposed MIS.

.
